# Women’s secure hospital care pathways in practice: a qualitative analysis of clinicians views in England and Wales

**DOI:** 10.1186/1472-6963-14-450

**Published:** 2014-10-01

**Authors:** Nadia Somers, Annie Bartlett

**Affiliations:** Department of Population, Health Sciences and Education, St George’s, University of London, Cranmer Terrace, London, SW170RE UK

**Keywords:** Women Mentally Disordered Offenders, Pathways of care, Continuity, Transition, Cost of care

## Abstract

**Background:**

In England and Wales women form a small but significant group within the wider, largely male, secure hospital population. Secure hospitals are designed to assess and treat individuals with both mental health problems and significant criminal behaviour. The theoretical approach to the care of secure hospital women is increasingly informed by a grasp of gender-specific issues. However, there is a lack of evidence on the adequacy of current structures and processes of care delivery.

**Methods:**

This qualitative study explores the nature and quality of care pathways for women in low and medium secure hospital beds by eliciting participants’ views of factors enhancing or impeding care. Beds are publicly funded and provided either by the National Health Service (NHS) or the Independent Sector (IS). Participants from both sectors were local experts (40 Consultant Psychiatrists, 7 Service Managers) who were well placed to describe their immediate health environment.

**Results:**

Evidence from the study indicates that participants were focused on the physical relocation of women to less secure conditions, even though many women do not readily achieve this.

Participants were alert to potential conflicts between ideal care and affordable care. Ideal care was compromised by the absence of suitable local services (beds or community placements), curtailed episodes of care and changes of care team. It was promoted by an awareness of the specific needs of women, continuity of care and support for teams unfamiliar with women’s needs.

**Conclusion:**

Future service design must address these challenges in care delivery, incorporating a better understanding of and response to the ways the system can echo women’s experiences of trauma and their negative attachment histories. Specifically, critical transitions in care must not be allowed to further reinforce the discontinuity, failure and rejection experienced by individual women earlier in their lives.

**Electronic supplementary material:**

The online version of this article (doi:10.1186/1472-6963-14-450) contains supplementary material, which is available to authorized users.

## Background

Regional and local secure hospital services in England and Wales for mentally disordered offenders have been developed since 1975 [1, 2] based primarily on the needs of men [3–6]. Recent guidelines for medium and low secure services aim to ensure national standards of care are met [7–9], but lack a gender dimension. Theoretical insights into the relevance of gender-specific issues (trauma histories and attachment styles) to the care of women in secure care has slowly emerged [5, 10–14]. However there is a dearth of evidence; outcome studies are small scale for example [15] or limited to recidivism [16]. The delivery of care via the current national system which utilises some 1625 women’s beds [17] is poorly described.

Caring for mentally disordered offenders, of both genders, involves movement through a multi-organisational system that includes the criminal justice system (police, court diversion schemes, prison), secure hospital care, step down and community (e.g. 24-hour supported accommodation) facilities [18]. In England and Wales, the National Health Service (NHS) and the Independent Sector (IS) (private and charity run organisations) provide secure services, which are purchased by NHS Commissioners. Routes in and out of secure care are complex and interdependent [1] and can be slow [19, 20].

The current qualitative study reports local, informed views of the nature and quality of pathways of care in women’s secure services. It adds to the existing literature by characterising problems encountered at transition points in care and contextualises these findings in the resources allocated to these high cost, low volume services. The data presented here represent part of the full analysis of interviews that revealed the nature of caring for women and women’s secure care pathways in practice. The current paper describes the latter, an understanding of the women’s care pathway in secure hospitals, which may differ from other health arenas. The study’s immediate practical significance is to inform the UK national service specification for these services, following the introduction of new commissioning structures in health and social care [21].

## Methods

This paper reports interview data from the national service evaluation of women’s secure care pathways [17, 19]. Service evaluation status was confirmed by the National Research Ethics Service and South West and St George’s Mental Health NHS Trust. Each participating NHS Trust approved the study through their audit or R&D departments.

### Participants

Participants were drawn from units identified in a wider study of secure hospital services to women in England and Wales (see [17, 19] for additional detail on units) and involved 96 of the 99 (19 of the 21 NHS and 40 of the 41 IS low secure units (LSU) and the 23 NHS and 14 IS medium secure units (MSU)) that took part in the national service evaluation [19]. All services were informed about the interviews when they agreed to take part in the study. We were able to obtain at least one interview, at the relevant security levels, for each NHS region. Nine IS providers were represented by at least one interview, five (of which four were not participating), all providing low secure beds, are not represented by an interview. Forty-seven interviews were conducted that represent 45 forensic services responsible for 56 (of a possible 99) secure units; 26 medium secure units (17 NHS, 9 IS) and 30 low secure units (15 NHS, 15 IS) and two Women’s Enhanced Medium Secure Services (a higher staff patient ratio than other medium secure services). Some services had both levels of security and eleven interviewees completed one interview for both tiers of security. In two units two interviews were completed for each unit.

Interviews were conducted with Consultant Psychiatrists, or Service Managers where they felt they had a fuller view of the clinical and business workings of the service or where the Consultant Psychiatrist was not available. This method is known as “elite interviewing” ([22], p105) where interviewees are picked for their experience and expertise, in this case knowledge of both patients and services; forty Consultant Psychiatrists and seven Service Managers or Nurse Directors took part.

### Interviews

The interviews were developed by the research team (Additional file 1), which included two Consultant Forensic Psychiatrists and two forensic mental health researchers [19]. Semi-structured interviews were conducted by asking respondents what were the Service’s strengths, weaknesses, opportunities and threats (SWOT framework) in four areas: clinical approach, care pathways, commissioning and financial issues. A SWOT framework was used as it is a helpful tool for understanding and planning services. SWOT comes from the field of management and business strategy, “…it invites decision makers to consider important aspects of their organisation’s environment and helps them organise their thoughts” ([23], p8). It is a tool to identify the positive and negative aspects, of an organisation, be they internal or external [24, 25].

In addition to the four topics to be discussed within the SWOT framework, three specific, open questions were added in anticipation of the national commissioning changes and to ensure the imposed framework did not limit responses: i) How does your local service fit into the national picture? ii) What would you change about your local service? iii) What would you change about the national picture?

Participants were given the interview framework (Additional file 1) in advance, at which point they were able to nominate a more suitable respondent if necessary. Due to the large numbers of interviews we aimed to collect, with participants from all over England and Wales, all interviews were conducted over the telephone by a Research Worker (NS) (n = 35) and a Forensic Psychiatrist (n = 12). Interviewers conferred about the level of prompting, which was kept to a minimum. The interviews were transcribed by the interviewer as they were being conducted, hence some quotes appear in a short hand form. The transcriptions were returned to the interviewee to review and amend if they wished. No major revisions were made to the transcripts. Interviews lasted between 20 minutes and one hour, with all respondents answering all questions. Interview subjects were assured of personal anonymity as well as anonymity of region and provider. Participants seemed engaged and eager to discuss issues relating to women in secure care.

### Analysis

A thematic analysis was conducted as described by Braun and Clarke [26] following the six phases of: familiarising oneself with the data, generating initial codes, searching for themes, reviewing themes, defining and naming themes and producing the report. The software programme NVivo [27] was used to aid and track manual analysis and features such as word frequency and other data examination facilities utilised. The analysis was conducted in the main by a research worker (NS) who was new to the field of forensic psychiatry, in order to minimise subjectivity. Initial coding on a subset of interviews was conducted independently by two Consultant Forensic Psychiatrists (one of whom, AB, is an experienced qualitative researcher) and finalised by consensus; the codes were expanded as additional data was examined and altered as iterative examination of the data developed by the research worker created a comprehensive analytic framework. Codes were categorised and, in turn, themes were allowed to emerge from the data. Codes and themes were not restricted to the SWOT framework and links across and between this interview framework were considered. As the analysis progressed a further sample of interviews were explored and cross-referenced by members of the research team and emerging themes discussed and considered.

## Results and discussion

Owing to the large quantity of data, the study is presented in two parts; the nature of caring for women mentally disordered offenders (MDOs) and, reported here, pathways in practice. In reality, these aspects are, and were in interview, intertwined. However, the natural ease in this separation reflects an empirical disjunction between respondents’ understanding of care needs and the functional processes of women’s pathways. We report here the practical, perhaps more bureaucratic, aspects of the pathway within the following themes: the nature of the “secure care pathway”, cost versus quality of care conflict, the false economy of rapid discharge, the peril of repatriation, gaps and blockages, competing concepts of continuity, fear and reluctance in receiving teams and access to community placements (Table 1). Quotes are included to illuminate the context and meaning of the themes.

**Table 1:** **Overarching themes and subthemes within “Pathways in practice”**

Overarching themes	Subthemes
The Nature of the “Secure Care Pathway”	Movement of women through tiers of security or out of services
	- Discharge facilitate
	Discharge delayed
Cost-quality of care conflict	The cost of caring for women
	- Nature of care is expensive
	- Cost is gender relevant
	- Determinants of bed purchase
	The false economy of rapid discharge
	- Discharge driven by cost
	- The negative effect of early discharge on recovery
	The peril of repatriation
	- Premature and disruptive discharge
	- Financially driven
Availability of Services	Gaps and blockages
	- Lack of service provision
	- Placements far from home
	- Provision of appropriate care in inappropriate security; continuity
	Access to community placements
	- Lack of step-down facilities and supported accommodation
	- Support and involvement in the community to prevent relapse
	- Broad conceptualisation of community (See Lists 2 & 3)
Attitudes to Care	Competing concepts of continuity
	- Continuity of treatment
	- Continuity of responsibility
	Fear and reluctance in receiving Teams
	- Stigma of women in secure services
	- Anxiety about receiving women from secure services
	- Outreach and supportive relationships

### The nature of the “Secure Care Pathway”

Participants’ descriptions of care pathways were understood in narrow terms and distinct from the commonly accepted, formal meaning. For them, care pathways meant the physical relocation of patients, not necessarily care delivery. In contrast, a care pathway, also referred to as integrated care pathways, within the NHS, is defined as:

“An integrated care pathway determines locally agreed multidisciplinary practice, based on guidelines and evidence where available for a specific patient/client group. It forms all or part of the clinical record, documents the care given, and facilitates the evaluation of outcomes for continuous quality improvement” ([28], p4).

A care pathway in the NHS is the set of defined activities and processes involved in a discrete aspect of care [29, 30]. For example, referral, assessment, intervention (usually aligned with National Institute of Clinical Excellence (NICE) guidelines) and discharge for a particular condition within a particular population. Emphasis may be on assessment and intervention phases as key processes involved in care.

Participants knew the study focused on the “care pathways of women mentally disordered offenders” but this was not defined for them. A handful of Consultant Psychiatrists enquired what this meant; they were asked to explain what it meant to them. One Consultant Psychiatrist held firmly that “pathways of care” was an unhelpful term, with no real meaning. Respondents were asked to describe a unit’s *intended* pathways of care. They were preoccupied with the physical movement of women out of their services, not assessment or intervention. Of the 50 responses to this question, only seven Consultant Psychiatrists said something about what treatment options and structure were within the service, of which all but one were based in the Independent Sector. Five more referred to their assessment process or acknowledged different care plans for those with personality disorders and mental illness. Without fail, every respondent described the source of admission and intended place of discharge.

Clinicians’ descriptions of care pathways suggested they were both linear and to lower rather than higher, or the same level of security. There was little acknowledgement that, in reality, patients move to higher security, for example from low to medium [19]. Clinicians were however describing *intended* pathways of care. This is at odds with the reality that a third of women are discharged to units of the same security level (ibid).

Secure services are required to detain mentally disordered offenders in the least restrictive level of security [11, 29–32] while also managing the risk they pose. The general preoccupation with movement down levels of security can be understood in that context. The principle of least restriction is particularly relevant to medium secure services where it forms part of the Department of Health Quality, Innovation, Productivity and Prevention (QIPP) Programme. This is, in turn, linked to the financial reality of units as they must demonstrate to commissioners they meet QIPP targets.

“Offering no thrills but fits in with QIPP; so we are keeping them moving into lower security. We focus on reducing risk and moving them on in timely fashion.” (IS)

Respondents focused on the point of discharge throughout the interviews; how discharge was facilitated or blocked. The next sections reveal how financial costs, attitudes to care and actual availability of services affected care pathways in practice.

### Cost-quality of care conflict

#### The cost of caring for women

Consultant Psychiatrists and Service Managers were acutely aware of the tension between cost and quality of care, believing women’s services were vulnerable to cuts in funding that reduce the quality of care. Respondents observed that caring for women was resource intensive, which they perceived to be greater than in men’s services due to the small numbers of women (creating concerns about unit size and viability), and high levels of both intensive observation (related to high incidence of self-harm [33] and staff absence, necessitating costly use of agency or bank staff.

“Expensive to run service and high level of observations. Very challenging, higher than average sickness rates and demand for staff for observations. So resource intensive.” (NHS)

“They [commissioners] don’t build in observations into budget and so problematic when increased” (NHS)

“We have a much reduced budget and how do we maintain quality standards when service is very resource intensive?” (NHS)

The gender-specific financial requirements were a source of frustration to a small number of units who felt their NHS Trust or wider Forensic Service was not responsive to these needs:

“Not acknowledging their special needs e.g. the new PD service is for men only. Hospital managers do not recognize complexity of women.” (NHS)

“Not investing enough in service. And treating like general service; underestimating staff input needs.” (NHS)

“They don’t commission services separately and so lose the essence of women’s service.” (NHS)

The extent to which individual units felt their commissioners considered quality of care varied. Many described having excellent working relationships with commissioners and case managers but most were acutely aware of financial and bed purchasing pressures.

“commissioners looking for cheapest providers, moving out of Private to NHS for money not clinical reasons; when we are not sufficiently staffed has implications for other patients.” (NHS)

“Commissioners need to look at outcomes more rather than cost. This emphasis on cheap service is a potential false economy.” (IS)

“Things are generally tight, which is a threat to patient care. E.g. [we] used to have art and drama therapy. Want more for less.” (IS)

In several units art and drama therapy were reported to be he first services to be cut (arguably essential in a population that may not be able to engage with traditional verbal psychological therapies). One unit could no longer continue their DBT (dialectical behavioural therapy for personality disorder) due to redundancies.

#### The false economy of rapid discharge

The cost-care conflict was at the forefront of respondent’s minds. In the context of Commissioner’s requests to reduce women’s length of stay, participants worried about the false economy of discharging women before they were clinically ready.

“financial situation- commissioning target reduces length of stay and so we need low secure service or step down that we can use. Commissioners are moving to a one in one out way rather than expanding.” (NHS)

“In the current financial climate there is a push for reducing length of stay. Women are high cost so pushing discharge too quickly; they [women] may not have all skills so ends in re-admission. Length of stay is captured but not looking at re-admission rates or community survival rates.” (NHS)

“The current climate is worrying due to the reduction in beds, length of stay and reduction of assertive outreach provision in general psychiatric service, resulting in quick and premature discharge before they are properly treated.

This results in relapsing with long-term consequence of treatment resistance etc. with increased re-admission rates and high need for rehabilitation, e.g. the referrals to x have increased…” (NHS)

Responding clinicians felt that progress made with women would be rapidly undone if they were prematurely discharged, discharged without sufficient transition time and planning and if they were discharged to unsuitable or insufficiently supported accommodation. Clinicians spoke about the shock of women leaving an intensive care setting, particularly when returning to the community.

“We provide a secure base then discharge suddenly: how can we manage that better. Outreach is constrained by funding.” (NHS)

“Three month disengagement package; we keep a bed for three months in case. Visit weekly for the first month. Helps the accepting team.” (NHS)

They emphasised the importance of a definite transition period, citing it as a major strength when available.

#### The peril of repatriation

Repatriation to the NHS from the Independent Sector was a common issue highlighted by the IS. This raised two key issues; disruption to care planning and treatment and the failure to use the resource of some IS services to provide continuity across several levels of security.

“Frustrating when they are moved in the middle of a placement when they have established relationships.” (IS)

“Premature discharge can derail treatment and rehab. Particularly with PD [personality disorder].” (IS)

The chief explanation given for repatriation was cost i.e. commissioners’ beliefs that the NHS was cheaper or that the NHS is the preferred provider, regardless of quality of care.

“Sometimes patients are taken back by the referrers to their local areas half way through their recovery. This decision is understandably mainly financially driven but it does have an impact on the patients’ journey to recovery” (IS)

“At times patients who are progressing well with treatment but do not require MSU are moved away by commissioners to other local LSUs, where treatment cannot be continued” (IS)

There was a sense of frustration from the Independent Sector, which provides 75% of women’s low secure beds [17]. Several Independent Sector providers were able to provide more than one level of the care pathway (a combination of or all of medium, low, step-down services) either on the same site or in close proximity and with good relationships between services. The IS lamented that they were rarely able to move women along what they considered to be faster or more streamlined, pathways. In addition, the IS, which collectively considered one of its main strengths as adapting quickly to the market and filling gaps in provision, was aware of a need for step-down and supported accommodation (discussed later) but felt rates of repatriation made the financial viability of such services questionable.

### Availability of services

#### Gaps and blockages

Within the secure pathway, for several medium secure units, within a number of regions, there was no geographically suitable low secure service. Gaps in provision meant women were prevented from moving on or were transferred to geographically distant locations.

“There is growth in medium secure units but not matched in low secure units.” (NHS)

“Some localities do not have dedicated LSU and patients need to be placed in out of area placements further away from home areas.” (NHS)

“Length of admission; women do get stuck longer than need to. Difficult to identify what care pathway, squeeze into what is available because of the lack of [low secure] provision.”(IS)

“NHS needs to wake up and provide local services for women. They are now OATs (out of area treatments).” (NHS)

For some regions, where low secure provision was absent, there was a sense that OATs were unavoidable. There was tension between women being placed far from home versus the potential benefits of receiving more specialised care.

This imbalance in provision has an impact on both directions of the pathway as highlighted by one respondent:

“Not enough low [secure] step up, so women are moving from PICU (psychiatric intensive care unit) straight to medium. Low [secure] provision needs assessing. Women in higher levels of security than is required for their needs, because there are not enough low secure beds.” (NHS)

Lack of provision can also result in women being kept at the wrong level of security. An alternative perspective came from several medium secure units. Limited or no low secure services resulted in medium secure units undertaking rehabilitation work. Women moved directly from medium security to the community. Participants thought this was beneficial to the patient.

“Patients stay in medium until supported tenancy as changing service would not be better” (NHS)

“Unique as it [the unit] offers long term stay on medium security levels but it functions as a low secure unit, through very robust relational security, creating a calm and safe environment.” (NHS)

“Medium can serve rehab function dealing with long term-why would there be a need to step down. Idea of linear pathways is absurd, but should keep [MDOs] in least level of security.” (NHS)

“Longer length of stay because not going to low secure so in higher security, but avoids destabilizing change.” (NHS)

The tension between achieving continuity of care and women being detained in undesirably high levels of security is conceptually important for how women’s secure care pathways are thought about and planned. It has long been a tenet of forensic practice that detention in the lowest level of security possible, bearing in mind the need to manage risk safely, is preferable [11]. Continuity of care, also judged to be a service design imperative, is, to judge from this data, both hard to achieve and, at times, privileged over this principle of minimal restriction. This reflects the de facto situation but cannot be assumed to have been as widely considered in service planning as is desirable.

#### Access to community placements

Successful discharge out of secure services depends on both an available community placement i.e. accommodation and a form of occupational/social engagement. Respondents described a lack of suitable provision in the community, step-down facilities and supported accommodation that sit outside secure services. This inability to access “the community” was thought to have a significant impact of length of stay in secure units as women became “stuck”.

“The only issue would be to enhance the pathway downstream to move people out and take more patients in.” (NHS)

“We are restricted in where we can send women; limit in what is available for complex women.” (NHS)

“Identifying a placement is an issue; there are small numbers of female only rehab placements-causes delays in low secure.” (IS)

Two major issues were highlighted. There was not enough supported accommodation and it was insufficiently varied to match women’s different capabilities. For example, there were several calls for partially supported and “homely” women-only accommodation, with staff who understood emotional crisis and self-harm. Respondents also wanted support for women in the community that would help them avoid readmission e.g. day centres that could act as crisis points, with well trained and supported staff.

“Community”, as invoked by participants was a broad concept related to the transition out of secure services (List 2). It not only referred to receiving mental health teams and supported and step-down accommodation on leaving secure services but also the need to access the community whilst in secure services, in order to acclimatise them to life outside and aid rehabilitation. This could be leave (sometimes difficult to obtain) and/or voluntary work projects (a result of local links and innovative practice). Accessing the community was a key consideration in moving women out of secure care but relied on a separate funding stream. As a result, respondents were keen to emphasise things that could be done to improve access to the community and thus the long-term rehabilitation of women leaving secure pathways. List 3 highlights these suggestions.

### List 2 Understanding “The Community”

**Participants’ use of the term “community”**

- Accommodation and supported accommodation/step down services.
- General mental health services and teams.
- Community access/participation: section 17 leave, volunteering, projects such as market stalls.

### List 3 Accessing the “The Community”

**Participant suggestions for improved access to the community**

- Filling gaps in accommodation provision; type and availability.
- Easily accessible information about available accommodation and services.
- Forensic outreach: support for MDO and receiving teams.
- Education of and support for local services.
- Commissioners funding “transitional pathway” for up to 6 months.
- Clear lines of responsibility for and access to funding streams.

### Attitudes to care

#### Fear and reluctance in receiving teams

Participants felt that non-forensic receiving teams lacked knowledge about the needs of this population and were anxious and unwilling to take these women onto their caseloads. The anxieties of receiving teams about taking “difficult” patients were cited as reasons for increasing length of stay by slowing down discharge.

“PCTs won’t accept because of self-harm, women difficult to manage so try and support placements where they go.” (NHS)

“Change people’s perceptions of it being so difficult. People don’t realize they move on and get better even though they are in secure, so when next present to acute there is stigma. (NHS)

“anxiety of the receiving team, focus on how they were rather than how the patient is now.” (NHS)

Women who were leaving secure services might face stigma. Participants thought support for receiving teams was crucial, even just being available on the telephone, to manage self-harm and the emotional demands of the work. Such support was critical to overcoming delays in leaving secure care but was limited by units’ funding or ability to provide informal support. Some units had access to established Forensic Outreach/Community Teams set up by their greater forensic service; other units were providing an informal and non-funded outreach service. Their task was to follow women up and provide them with a transitional link between the old and new service, as well as for staff in the receiving service. Forensic outreach services helped speed up the discharge process because positive relationships with receiving teams allowed experienced forensic practitioners to advise and allay the fears of receiving teams.

“Will offer receiving teams advice. Go out twice in first month to check women, on existing budget.” (NHS)

“We have good links with step down services and work with those teams when the patients experience anxieties.” (NHS)

The IS reported difficulties with communication and relationships with receiving teams. IS units rely on commissioner case managers to identify and maintain contact with receiving units that are geographically distant; this input varies both individually and by region.

#### Competing concepts of continuity

Continuity of care in these data was a multi-faceted concept, involving both continuity of responsibility and continuity of treatment [31, 34]. Continuity of care referred to the consistency of staff within the ward and across wards within the service, the maintenance of treatment approaches and clearly defined boundaries (i.e. relational security), well-planned transition periods, without bureaucratic disruptions and communication with receiving teams.

“[We] do not use agency staff and have internal bank cover but still these staff would not know patients very well and there is at times disruption to continuity of care.” (IS)

“[We have] continuity of care as I also work in the community. Good relationships with community teams mean it is easier to transfer out.” (NHS)

“Have fairly self-contained levels and units within the service. Same staff looking after transition phases. Gives patients a lot of incentive to move on and continuity of care.” (IS)

The importance of continuity to participants was evident from the desire to provide a complete pathway i.e. all tiers of security ideally on one site, or within one forensic service. This was particularly highlighted by the IS who thought it would enhance communication, consistent approaches to care and best use the experience and knowledge of the care team.

“[We have a] range of services on one site. We can take acute phases of illness and move through the service into rehab and being prepared for discharge into community.” (IS)

The benefits of pre-discharge or rehabilitation wards within an existing unit, were discussed by both the NHS and IS sectors. These wards treat and move women on, creating continuity in staff and clinical approach as well as something for women to “aim for”. They also offer reassurance to potentially anxious receiving services.

“pre discharge very helpful; we can say to the community they have been living in open environment.” (NHS)

The desire to have all sections of the pathway to hand or to create a sense of progress (pre-discharge wards) points not only to the need to have treatment continuity but also to the wish of units to retain clinical responsibility. This did not necessarily mean the care team remained the same, although when it was available, it was cited as a major strength. Rather, continued responsibility means a provider (NHS and IS) can move women on without having to engage with outside teams, gatekeeping, funding issues, bed waits and can control transitions. Maintenance of responsibility contributes to continuity of care by enabling relationships between internal units and staff and swift communication.

Continuity of responsibility was disrupted at the interface between funding bodies of secure and non-secure services. The change in funding streams, from forensic commissioning to Primary Care Trusts (PCT) funding, ^a^created delays detrimental to the momentum of care and uncertainty and anxiety in the women. Securing funding was time-consuming and destabilising. The wish was for continuity of responsibility to go beyond transitions within secure services [31] and smooth the boundary between secure and general mental health services. Several responding clinicians said that specialised commissioners should fund the first few months out of secure care (3–6 months).

“Commissioning care pathways rather than care spells. Following all the way through to community; if maintain women in same environment it allows them to change. Commission right into community.” (NHS)

“How commissioners purchase beds rather pathways of care. Would give patient and us stability.” (IS)

Women trying to escape difficult situations related to family, relationships and substance abuse may need to leave their home region. Acquiring rehabilitation funding from “home” PCTs to these new communities was described as “notoriously difficult”. The IS, with a national intake, reported this more frequently than the NHS.

### Limitations

The aim of the present study was to provide a nuanced overview of how women’s secure care pathways should, could and do work. Its national remit meant sacrificing a degree of depth that might have been achieved in longer face to face data collection. Telephone interviewing did enable us to reach a large number of geographically disparate respondents. A limitation, compared to face-to-face interviews, was the unavailability of some visual, social cues, which facilitate clarification, prompting and a greater understanding of the context of the service. We were also unable to record the interviews and some information or meaning may have been lost in the immediate transcription, as the interviewer simultaneously processed several types of information. The current study used respondent validation (review, amendment and approval of interview transcripts) before text analysis for this reason. The study would have been enhanced by respondents’ review of themes emerging from the data but this was incompatible with the project’s resource management. This is only one of several ways to check credibility; we did use peer examination, which was conducted throughout the analysis process [35].

## Conclusion

Health funding in the UK is being severely reduced [36, 37]. Respondents reported a tension between moving women out of long term, expensive, secure hospital care and the clinical need to ensure consistent, high quality and resource intensive care that might facilitate successful, long-term rehabilitation. Clinicians’ concepts of a pathway concerned moving women out of services. They described several factors that delay transfer and some that speed it up. Rapid discharge or repatriation from the independent sector to the NHS was considered to disrupt treatment and, in the long term, to result in avoidable readmission.

Respondents’ insights have revealed a conflict between the actual and the preferred, clinical pathway. Continuity of care was jeopardised by the reality of the care system. Without service user views on the subjective importance of continuity of care and the pros and cons of specific elements of the pathway, the significance of this finding cannot be fully assessed. However, the system as described is not functioning as intended. Further research is also required to go beyond the current qualitative work and elucidate the *extent* to which the pathway is not functioning in the described ways and the associated impact on psychological and reoffending outcomes.

The individual histories of women in secure hospital services are characterised by early trauma and pathological attachment structures [38–40] rendering them particularly sensitive to feelings of abandonment and rejection, encapsulated in high levels of borderline pathology in this population [19]. The discharge of some women from these services appears overly driven by financial and commissioning pressures. If the respondents’ views are well founded, the recommended, gendered approach [12, 13] to this population’s clinical needs of continuity of care (within a service and of individual staff) is compromised. Sudden and/or premature discharge combined with insufficient and changeable staffing, may resonate with women’s individual experiences which, if ignored, may undermine their progress. Potentially self-punitive perceptions, born of chronically low self–esteem, may stem from remaining in unnecessarily high levels of security. Concomitant, limited opportunities to gain confidence and responsibility in the community may jeopardise long term rehabilitation in the community. This theoretically plausible understanding needs greater empirical exploration. However, future service design cannot be oblivious to this analysis of the system of care [41, 42].

## Endnote

^a^PCTs were abolished by the Health and Social Care Act 2012. Their function of commissioning local general health care in a small geographical area has been taken over by a Clinical Commissioning Group.

## Authors’ information

Nadia Somers: Research Worker SGUL (Current role: Trainee Clinical Psychologist, Royal Holloway University of London).

Annie Bartlett: Reader and Honorary Consultant in Forensic Psychiatry SGUL & Clinical Director (Jnt) Offender Care Central North West London NHS Foundation Trust.

## Electronic supplementary material

Additional file 1: **Interview Template.** (DOCX 16 KB)
