# Supplementary material for: Women’s secure hospital care pathways in practice: a qualitative analysis of clinicians views in England and Wales
Source: BMC Health Serv Res. 2014 Oct 1;14:450. doi: 10.1186/1472-6963-14-450 (PMC4263065; doi:10.1186/1472-6963-14-450)
Supplement: Supplementary file 1 — Additional file 1: Interview Template. (DOCX 16 KB) [file 12913_2013_3536_MOESM1_ESM.docx]

Interview

**Date:**

**Name of interviewee:** **Role:**

**Name of Research Worker:**

**Length of interview:**

- **Please describe your current service for women.**
- Diagnostic/risk profile
- Staffing levels by discipline
- Buildings (old building/separate from men’s unit)
- Intended pathways of care

**SWOT**

- **Clinical Approach**
- Strengths:
- Weaknesses:
- Opportunities:
- Threats:
- **Pathways of Care**
- Strengths:
- Weaknesses:
- Opportunities:
- Threats:
- **Commissioning**
- Strengths:
- Weaknesses:
- Opportunities:
- Threats:
- **Financial**
- Strengths:
- Weaknesses:
- Opportunities:
- Threats:
- **How does your local service fit into the national picture?**
  - **What would they change about their local service?**
  - **What would they change about the national picture?**
